# Blunt Trauma to the Abdomen Leading to Incarcerated Ventral Hernia: A Case Report

**DOI:** 10.7759/cureus.89787

**Published:** 2025-08-11

**Authors:** Shikha Tiwari, Manoj Sonkar, Naveen K Patel, Rajat Kumar Singh, Baby Sharma

**Affiliations:** 1 General Surgery, Ganesh Shankar Vidyarthi Memorial (GSVM) Medical College, Kanpur, IND

**Keywords:** abdomen ventral hernia, acute care surgery and trauma, complicated hernia, high velocity blunt trauma, small bowel obstruction

## Abstract

Traumatic abdominal wall hernias are a rare complication of blunt abdominal trauma, with only a small percentage of cases reported in trauma centers. This report discusses a unique case of a 36-year-old female who developed a de novo incarcerated ventral hernia in the left iliac fossa, following a road traffic accident. The herniation involved a segment of small bowel entrapped in the defect, along with mesenteric tears and hemoperitoneum. The patient underwent emergency laparotomy, during which bowel viability was confirmed and primary repair of the defect was performed without bowel resection. The case emphasizes the importance of timely diagnosis and intervention in complicated trauma-related hernias to avoid further deterioration of the condition, like strangulation.

## Introduction

Ventral hernias most commonly occur in individuals with a weakened abdominal wall, including both fascia and muscle layers. This weakening is often due to prior laparotomies [1], although congenital causes such as umbilical hernias also exist and may resolve spontaneously. Various predisposing factors increase the risk of ventral hernias, including smoking, abdominal distension from ascites, pregnancy, heavy physical activity, chronic constipation or coughing, and connective tissue disorders [2].

Blunt abdominal trauma is frequently encountered in emergency settings, yet the occurrence of a traumatic abdominal wall hernia (TAWH) remains rare, with a prevalence of about 1% even in highly specialized trauma centers [3]. The low incidence may be due to the critical nature of many trauma patients, where less obvious injuries are missed during the initial management of life-threatening conditions. However, associated injuries like pelvic and rib fractures, lumbar vertebra fractures, abdominal wall hematomas, and abrasions should arouse an index of suspicion for such cases. This report presents a rare case of TAWH with features suggestive of bowel obstruction, successfully treated through emergency laparotomy.

## Case presentation

**Figure 1:**
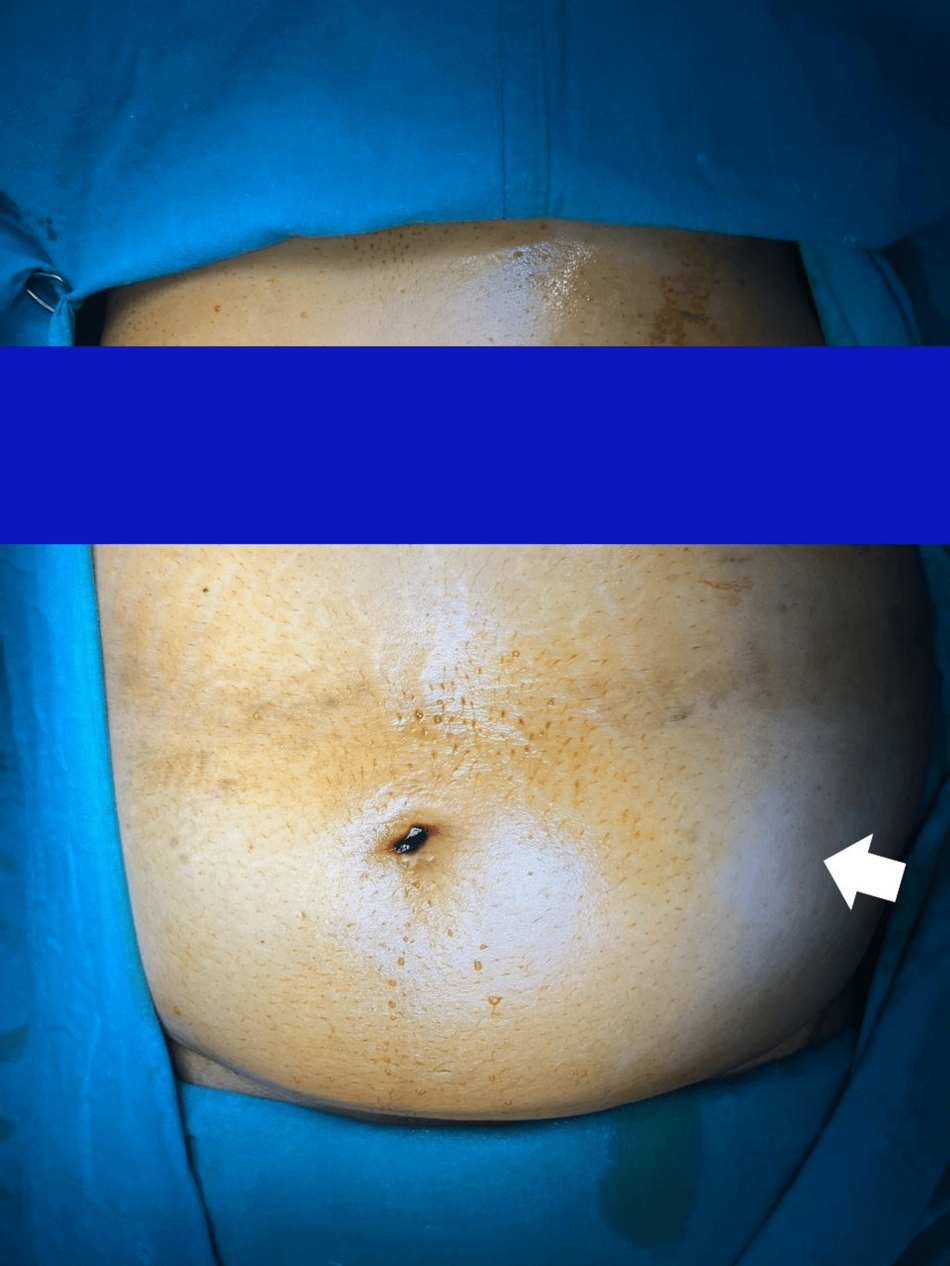
Preoperative image showing a bulge in the left iliac fossa and lumbar region. Solid white arrow indicates the bulge of the hernial sac in the left iliac fossa extending into the left lumbar region.

A 36-year-old female presented with abdominal pain and distension, one day following a road traffic accident due to the collision of a rickshaw, on which the patient was seated, with a four-wheeler, resulting in blunt abdominal trauma. She was obese, pale, and had an irreducible asymmetrical bulge in the left iliac fossa with ill-defined margins (Figure 1). Bowel sounds were absent. Aside from minor facial abrasions, no other major injuries were noted.

**Figure 2:**
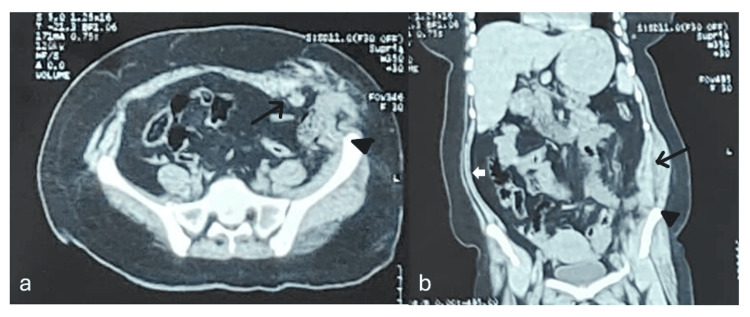
Contrast-enhanced CT (CECT) of the pelvic region with iliac blades in view, showing the level of abdominal wall rupture. (a) Axial view. (b) Sagittal view. Black arrow: Disruption in the abdominal wall on the left side. Black solid arrowhead: Left iliac crest. White arrow: Normal smooth outline of the right abdominal wall, in contrast to the ruptured left side.

**Figure 3:**
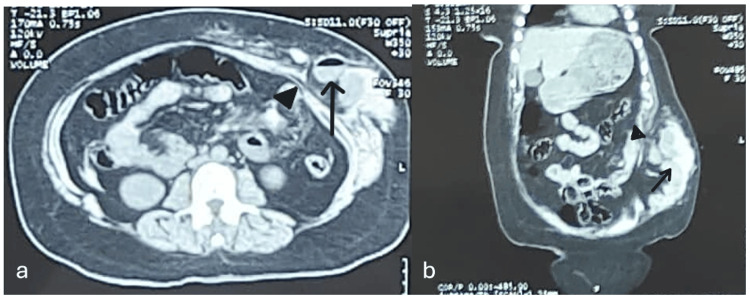
Contrast-enhanced CT (CECT) of the whole abdomen showing a defect in the musculature of the anterior abdominal wall with herniation of small bowel loops. (a) CT image of abdomen in axial plane, solid black arrowhead - tear in muscle layer, black arrow - loops of small bowel with air-fluid level. (b) CT image of abdomen in sagittal plane, solid black arrowhead - tear in muscle and fascia of abdominal wall, black arrow - loops of small bowel.

A contrast-enhanced computed tomography (CECT) scan revealed disruption of abdominal wall musculature and fascia in the left iliac fossa (Figure 2), and herniation of small bowel loops through the defect (Figure 3). The patient was hemodynamically stable, with a hemoglobin level of 9.9 gm/dL (normal range = 12.0-15.5 g/dL) and normal leukocyte count (4,000-12,000 cells per microliter).

**Figure 4:**
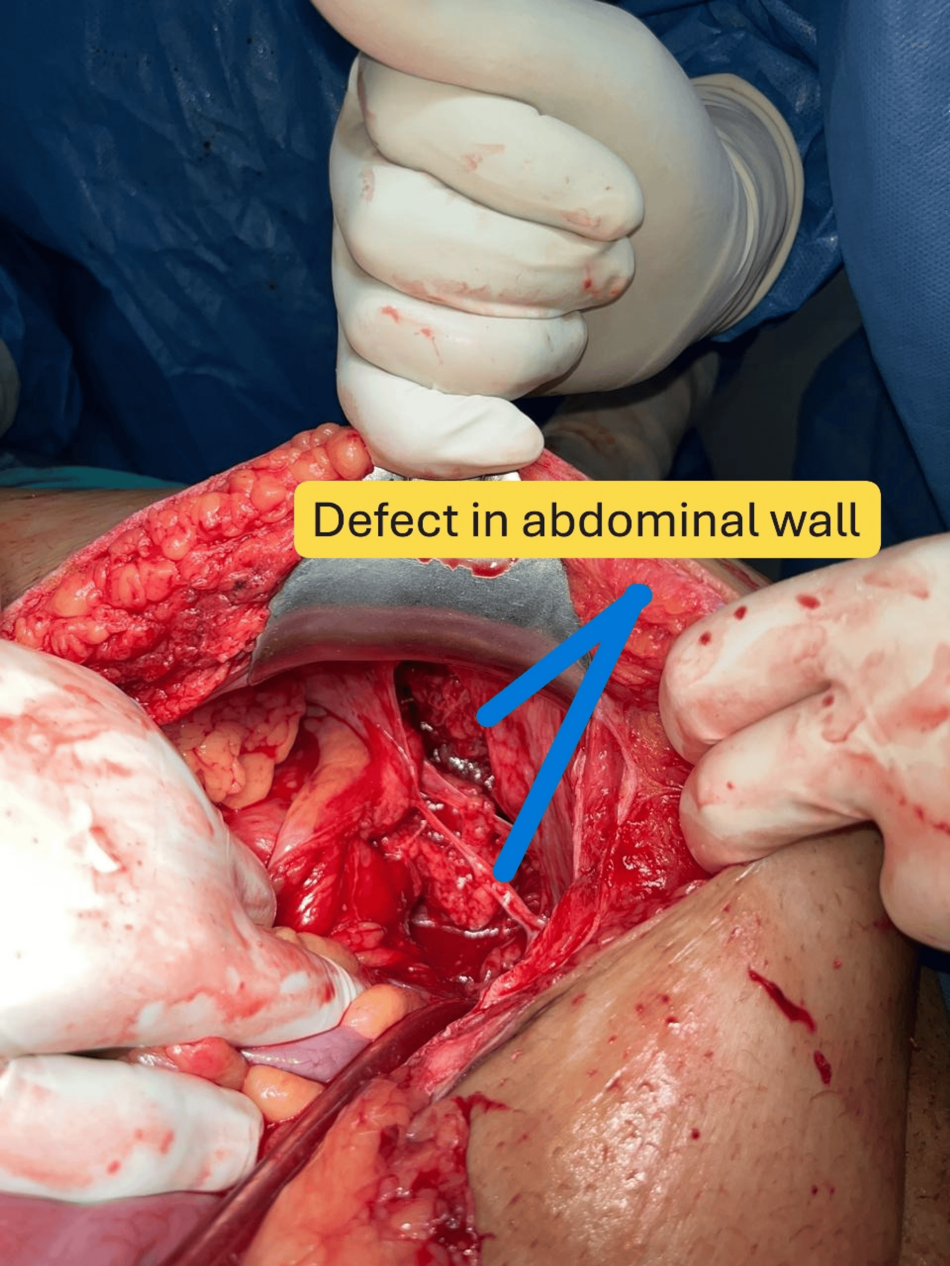
Defect seen in the left iliac fossa region after laparotomy, showing peritoneal, muscular, and fascial breach.

Following appropriate preoperative preparation - including prophylactic antibiotics, fluid resuscitation, and insertion of gastric and urinary catheters - the patient underwent exploratory laparotomy. Approximately 700 mL of fresh blood was drained. A 30 cm segment of small bowel, approximately 90 cm distal to the duodenojejunal junction, was found entrapped within a 7 × 5 cm abdominal wall defect (Figure 4). The defect extended to the retroperitoneal plane, exposing the psoas muscle.

**Figure 5:**
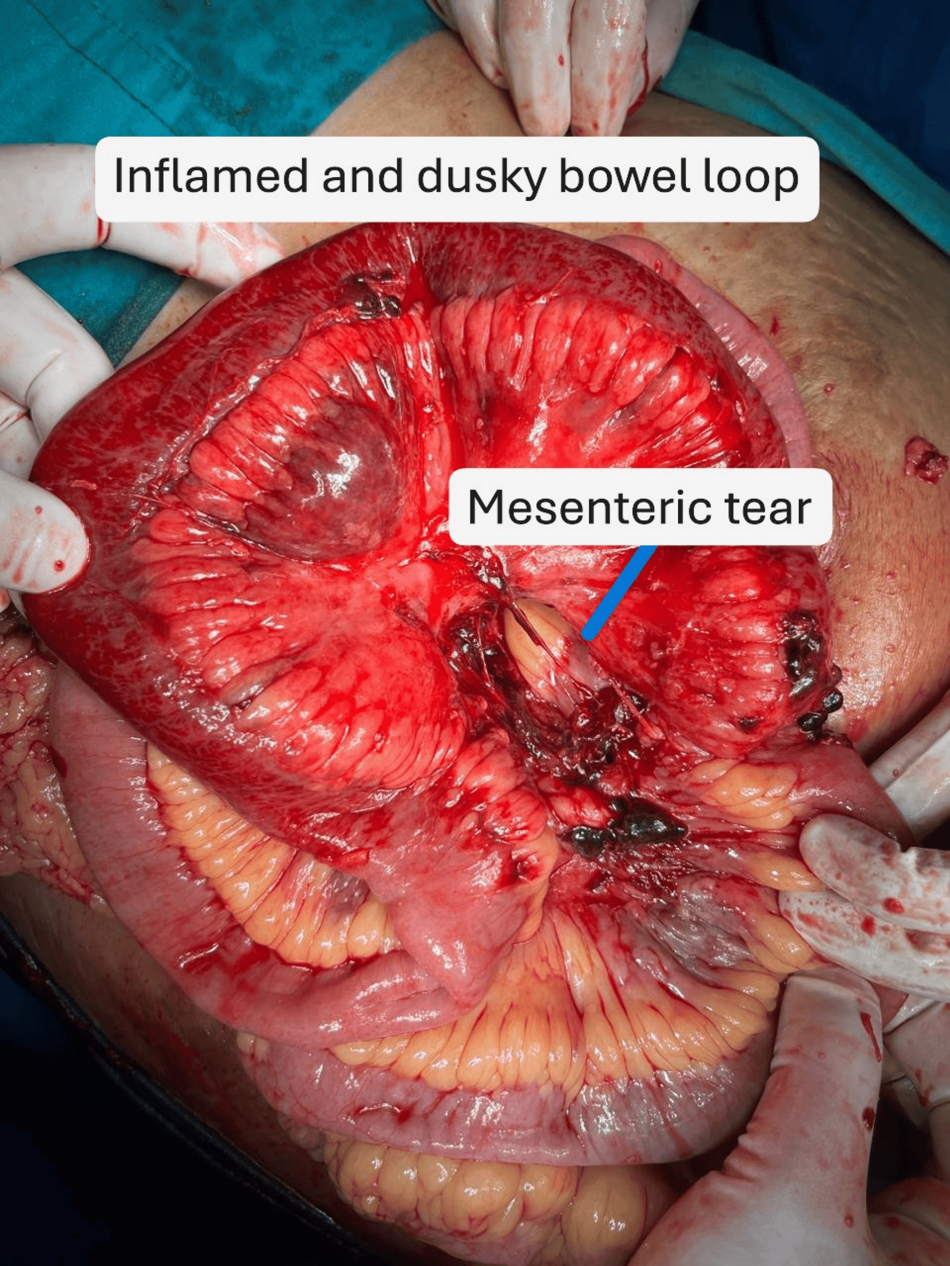
Image showing a mesenteric tear and an inflamed bowel loop that was herniated.

The herniated bowel appeared dusky and inflamed, with mesenteric tears measuring around 5 and 3 cm (Figure 5). Warm saline-soaked mops and supplemental oxygen were applied for 15 minutes, resulting in a return of pink coloration, indicating viability. Consequently, bowel resection was avoided. No abnormalities were found in other intra-abdominal organs.

The mesenteric tears were repaired using non-absorbable silk 2-0 sutures. The abdominal wall defect was closed in two layers: the muscular layer with non-absorbable Prolene 2-0 sutures and the peritoneal layer with absorbable monofilament 2-0 sutures. Two drains were inserted-one in the pelvic cavity and another in the retroperitoneal space. The rectus sheath was closed with PDS 1-0, and the skin was closed in layers with a negative suction drain (Minivac) in place.

The first dressing on postoperative day 2 revealed no signs of surgical site infection. Oral feeding was resumed the same day and was well tolerated. The patient was discharged on postoperative day 5 with stable vitals and remained complication-free on follow-up after two months.

## Discussion

The patient had multiple risk factors for ventral hernia development, including obesity, previous pregnancies, and trauma. The findings of mesenteric tears and compromised bowel viability in this patient align with reports by Netto et al., who observed that frequent visceral injuries were present in such presentations [3]. While solid organ injuries (e.g., liver, spleen, kidney) are more commonly seen in blunt abdominal trauma [4], TAWH remains a rare but significant finding. Diagnostic imaging, especially Extended Focused Assessment with Sonography for Trauma (EFAST) and CT scans, is crucial in evaluating such injuries [4,5]. TAWH is typically defined by two criteria: first, immediate onset after trauma without associated skin penetration (as seen in bowel evisceration), and second, persistent physical signs of trauma at presentation [6]. The sudden onset of symptoms following a traumatic event, absence of hernia in the past, and confirmation on radiological investigations suggest that this patient aligns with the existing criteria set by the existing literature. Wood et al. classified TAWHs into three categories based on the mechanism of injury [7]. Type I involves muscle tears due to sudden increases in intra-abdominal pressure from high-energy trauma (e.g., motor vehicle accidents, falls), with frequent injury to intra-abdominal organs. This patient's injury falls into this category, commonly affecting the flank or upper quadrants, and additionally, the CT findings of a 7 × 5 cm defect with bowel herniation and hemoperitoneum support this categorization. Type II is caused by low-energy, focused trauma (e.g., bicycle handlebar), known as a handlebar hernia. These are more frequent but less likely to be associated with intra-abdominal injuries. Type III results from deceleration injuries. 

Although conservative management and delayed repair are sometimes advised, patients presenting with signs of bowel incarceration, obstruction, or significant hemoperitoneum require prompt surgical intervention [8]. The use of mesh for repair in such cases is debatable, especially in emergency settings where the risk of infection is higher. Studies such as those by Yadav et al. emphasize the increased risk of postoperative infection when mesh is used in emergency settings, particularly in the presence of bowel handling or hemoperitoneum [8]. Biological meshes, derived from human or animal tissues and processed to remove antigenic components while preserving the extracellular matrix, are useful for complex abdominal wall repairs where synthetic mesh is contraindicated. But, being very expensive and having long-term durability, their routine use is limited. Therefore, primary repair using non-absorbable sutures is generally preferred. The decision to avoid mesh placement and instead opt for primary suture repair in two layers (muscle and peritoneum) thus follows the current surgical guidelines that caution against synthetic mesh use in contaminated or emergency fields.

A multicentric, retrospective study by Harrell et al. evaluated 281 patients with TAWH, of which 176 (62.6%) had undergone operative hernia repair. Among those who underwent surgical intervention, 157 (89.3%) were repaired primarily, while others had a delayed repair. Bowel injury was found in 95 cases (33.8%) [9]. The findings in our patient closely align with the findings of this study.

## Conclusions

TAWHs are a rare clinical entity most often presenting in the lumbar region. The reported incidence of traumatic hernias is currently low, as they are overlooked in the oversight of more life-threatening injuries. But regular and timely secondary surveys may help detect such injuries earlier. While conservative management may suffice in some cases, emergency laparotomy is indicated in patients with incarceration, obstruction, or hemodynamic instability. Timely diagnosis and surgical intervention are essential to ensure favorable outcomes.
